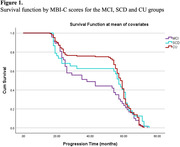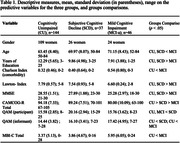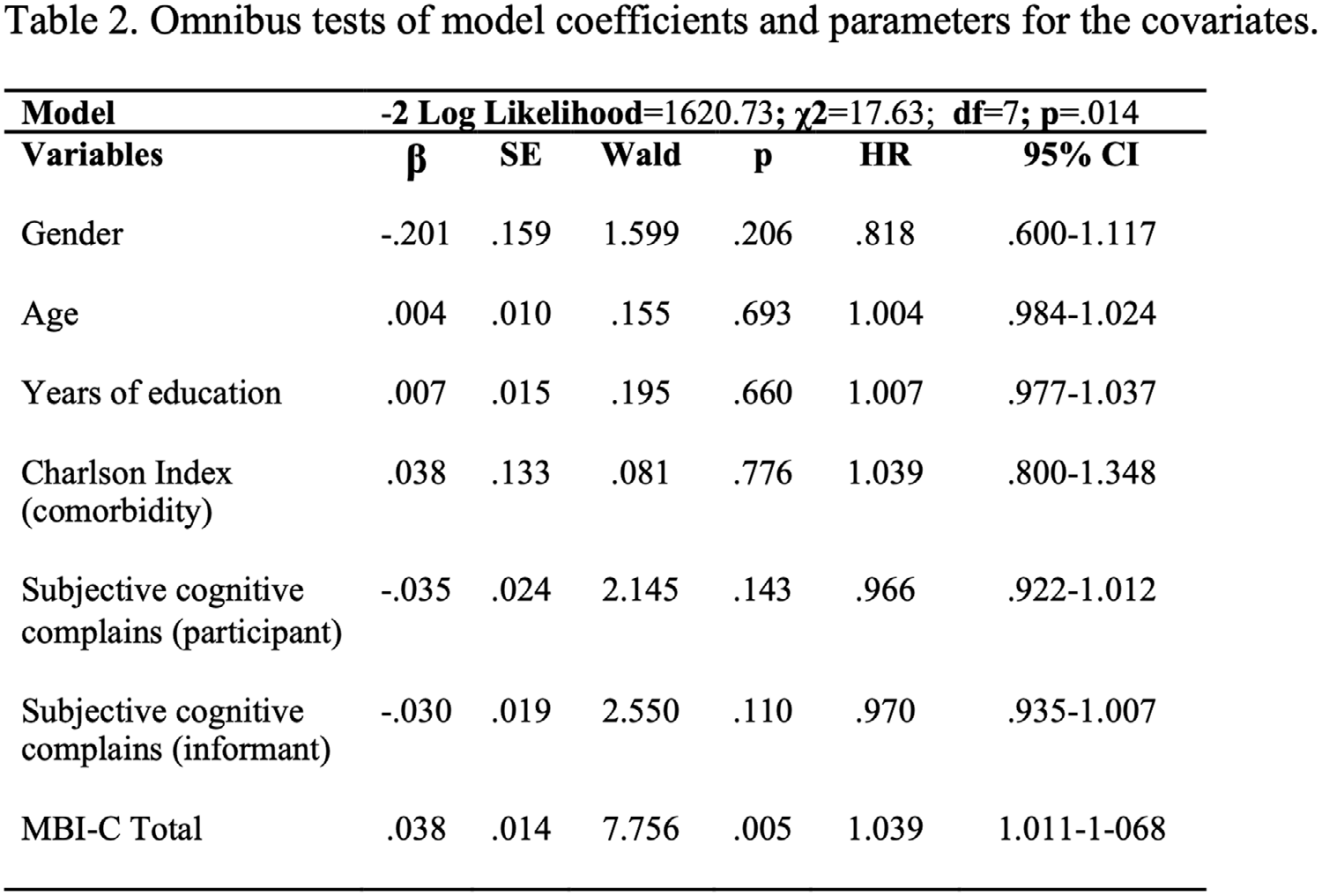# Can MBI‐C scores predict time‐to‐cognitive worsening in CU, SCD, and MCI participants? Evidence from the CompAS study

**DOI:** 10.1002/alz.091582

**Published:** 2025-01-03

**Authors:** Sabela Carme Mallo, Onésimo Juncos‐Rabadán, Cristina Lojo‐Seoane, Lucía Pérez‐Blanco, Alba Felpete, M. José Moreno‐Carretero, Águeda Rojo‐Pantoja, Ana Nieto‐Vieites, Arturo X. Pereiro Rozas

**Affiliations:** ^1^ Deparment of Developmental and Educational Psychology, University of Santiago de Compostela, Santiago de Compostela Spain; ^2^ Department of Developmental and Educational Psychology, University of Santiago de Compostela, Santiago de Compostela Spain; ^3^ Applied Cognitive Neuroscience and Psychogerontology group, Health Research Institute of Santiago de Compostela (IDIS), Santiago de Compostela Spain; ^4^ Galicia Sur Health Research Institute (IISGS), Vigo, Pontevedra Spain

## Abstract

**Background:**

The role of neuropsychiatric symptoms (NPS) as early markers of cognitive impairment progression along the cognitive impairment continuum needs further research. Our objective was to estimate to which extent NPS, measured with the Mild Behavioral Impairment Checklist (MBI‐C), predict time‐to‐cognitive‐worsening during follow‐up in a sample of older adults recruited from primary care centers while controlling for other related variables (i.e., age, gender, years of education, comorbidity, and cognitive complaints).

**Method:**

A multivariate Cox proportional hazard regression model was performed using the ‘Enter method’. Two hundred twenty‐six participants from the Compostela Aging Study (CompAS) were included in the analyses (none censored). Participants were diagnosed at baseline as Cognitively Unimpaired (CU = 144), Subjective Cognitive Decline (SCD = 37), and Mild Cognitive Impairment (MCI = 45) (Table 1). The event was defined as stability or worsening in cognitive status over a follow‐up of 17‐75 months, during which three evaluations were performed. Stability was considered when the diagnosis remained stable and worsening when progression to MCI or dementia occurred. The groups at baseline (CU, SCD, MCI) were entered into the model as strata. MBI‐C total score at baseline was introduced as a covariate and adjusted by age, gender, years of education, comorbidity, and SCCs from participants and informants.

**Results:**

The tested model was statistically significant [overall significance: *‐2 Log Likelihood* = 1620.73, *χ^2^* = 17.63, *df* = 7, *p* < .014]. MBI‐C total score was the only significant variable [*β* = .038, *SE* = .014, *Wald* = 7.77, *p* < .005]. Its corresponding hazard ratio was 1.039 (95% CI 1.011‐1.068), indicating that for one unit increase in the MBI‐C total score, the risk of worsening increases by 1.039 for a month (Table 2).

Figure 1 shows the survival function using covariates for each group. The greatest differences were observed in the time interval 20‐60 months, and especially between the MCI and CU. SCD curve remained stable during that period and was more similar to the MCI curve.

**Conclusion:**

Our results indicated that MBI‐C can significantly predict time to worsening along the cognitive impairment continuum. Further, differences between MCI and CU participants' trajectories were especially pronounced around 40‐60 months, with SCD falling between the two groups.